# The Positive Effects of *Grifola frondosa* Heteropolysaccharide on NAFLD and Regulation of the Gut Microbiota

**DOI:** 10.3390/ijms20215302

**Published:** 2019-10-24

**Authors:** Xin Li, Feng Zeng, Yifan Huang, Bin Liu

**Affiliations:** 1 National Engineering Research Center of JUNCAO Technology, Fujian Agriculture and Forestry University, Fuzhou 350002, China; lixin1229@hotmail.com; 2 College of Food Science, Fujian Agriculture and Forestry University, Fuzhou 350002, China; 3 College of Animal Science, Fujian Agriculture and Forestry University, Fuzhou 350002, China

**Keywords:** *Grifola frondosa*, NAFLD, gut microbiota

## Abstract

Non-alcoholic fatty liver disease (NAFLD) is a major public health problem in many countries. In this study, the ability of *Grifola frondosa* heteropolysaccharide (GFP) to ameliorate NAFLD was investigated in rats fed a high-fat diet (HFD). The molecular mechanisms modulating the expression of specific gene members related to lipid synthesis and conversion, cholesterol metabolism, and inflammation pathways were determined. The components of the intestinal microflora in rats were analyzed by high-throughput next-generation 16S rRNA gene sequencing. Supplementation with GFP significantly increased the proportions of *Allobaculum*, *Bacteroides*, and *Bifidobacterium* and decreased the proportions of *Acetatifactor*, *Alistipes*, *Flavonifractor*, *Paraprevotella*, and *Oscillibacter*. In addition, *Alistipes*, *Flavonifractor*, and *Oscillibacter* were shown to be significant cecal microbiota according to the Spearman’s correlation test between the gut microbiota and biomedical assays (|r| > 0.7). Histological analysis and biomedical assays showed that GFP treatments could significantly protect against NAFLD. In addition, *Alistipes*, *Flavonifractor,* and *Oscillibacter* may play vital roles in the prevention of NAFLD. These results suggest that GFP could be used as a functional material to regulate the gut microbiota of NAFLD individuals.

## 1. Introduction

A high-fat diet (HFD) is a risk factor for a range of diseases, including lipid metabolism disorders (LMDs), non-alcoholic fatty liver disease (NAFLD), and type-2 diabetes (T2D) [1,2]. NAFLD is currently the most prevalent chronic liver disease worldwide and has become a major public health problem in many countries [2,3]. The majority of NAFLD patients do not recover from this condition; rather, the disease progresses to non-alcoholic steatohepatitis (NASH), hepatocyte fibrosis, cirrhosis, or even hepatocellular carcinoma which are responsible for most deaths worldwide [4]. However, the factors involved in the induction and progression of these disorders are complex and not fully understood [5].

NAFLD is usually considered to be the hepatic manifestation of metabolic syndrome [6]. However, some clinical data have shown that the composition and characteristics of the intestinal flora in patients with metabolic disorders differ from those in normal populations [7,8]. For example, a significant correlation has been shown between the relative abundance of intestinal flora and hepatic dyslipidemia and inflammation [9,10]. Currently, a lot of drugs which have been tested in clinical trials could not provide satisfactory results in terms of therapeutic efficacy and side effect. In addition, only a few of these drugs target the intestinal flora in NAFLD. Therefore, it is necessary to search for a novel drug or natural compound without any side effects and find out how the compound relates to the intestinal flora in the treatment of NAFLD. In recent years, many polysaccharides with antihyperlipidemic, hepatoprotective, and antioxidant activities have been studied as dietary supplements or functional foods in the treatment of NAFLD [11,12,13,14]. However, it is generally known that polysaccharides are biological macromolecules and cannot be directly absorbed and utilized [15]. Fortunately, a previous study has reported that many polysaccharides could be utilized by the intestinal flora [16]. Therefore, the intestinal flora may be a major factor in the mechanisms of the polysaccharides and the relationship between the effect of polysaccharides and intestinal flora in the treatment of NAFLD still remains to be further studied.

Polysaccharides come from a wide range of sources, including animals, plants, fungus, and others [17]. *Grifola frondosa* is an important medicinal fungus in China and Japan, with diverse bioactive metabolites [18]. The polysaccharide extracted from *Grifola frondosa* has various biological and pharmacological activities, such as antioxidant, anti-inflammatory, immune-regulating, and antitumor activities [19,20]. Although its consumption is generally accepted to be beneficial for health, its mechanisms and relationships with NAFLD and the gut microbiota have not been completely investigated. Therefore, this research sought to assess the potential of *Grifola frondosa* heteropolysaccharide (GFP) supplementation in ameliorating NAFLD in Wistar rats fed HFD and to investigate the relationship of this mushroom with NAFLD-associated parameters and the gut microbiota.

## 2. Results

### 2.1. Effects of GFP on Body Weight and Biochemical Values of NAFLD Rats

**Figure 1:**
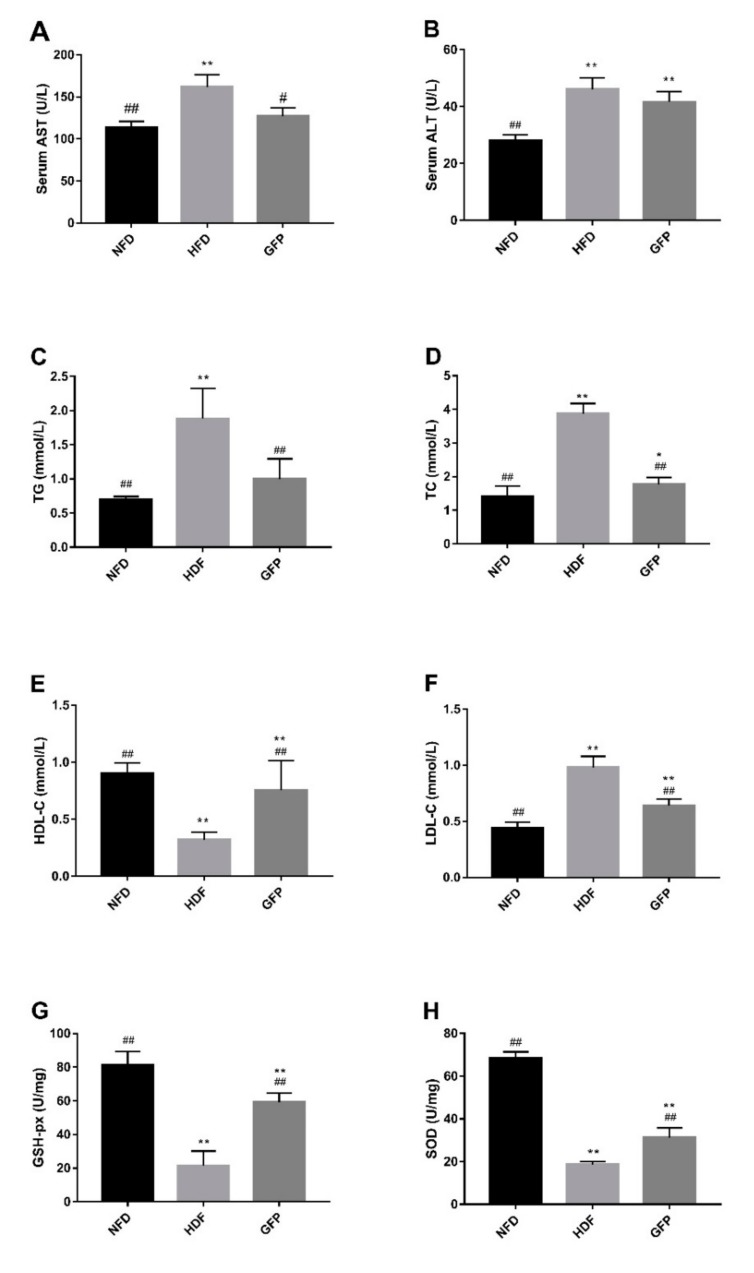
Effects of GFP on liver protection and lipid levels of non-alcoholic fatty liver disease (NAFLD) rats: (**A**) serum aspartate transaminase (AST); (**B**) serum alanine transaminase (ALT); (**C**) lipid triglycerides (TG); (**D**) total cholesterol (TC); (**E**) high-density lipoprotein cholesterol (HDL-C); (**F**) low-density lipoprotein cholesterol (LDL-C); (**G**) liver glutathione peroxidase (GSH-px); and (**H**) liver superoxide dismutase (SOD). Values are presented as the mean ± SD (*n* = 5). Differences were assessed by ANOVA and are denoted as follows: * *p* < 0.05 versus the NFD group; # *p* < 0.05 versus the HFD group; ** *p* < 0.01 versus the NFD group; and ## *p* < 0.01 versus the HFD group.

A steep increase was observed in the bodyweights of all rats (Table 1), with the weights of HFD rats increasing most significantly after four (*p* < 0.05) and eight weeks (*p* < 0.01). Animals in the GFP group showed obviously lower body weights than those in the HFD group after four and eight weeks (*p* < 0.01). The bodyweight gain of animals in the GFP group (154.33 ± 32.75 g) was significantly lower than that in the HFD group after eight weeks (*p* < 0.01), while there was no significant difference between the GFP and control diet (NFD) group.Increased alanine transaminase (ALT), serum aspartate transaminase (AST) lipid triglycerides (TG), and total cholesterol (TC), and low-density lipoprotein cholesterol (LDL-C) concentrations and decreased liver glutathione peroxidase (GSH-Px), and liver superoxide dismutase (SOD) and the levels of high-density lipoprotein cholesterol (HDL-C) concentrations were the primary features of NAFLD status of rats in the HFD group [21]. However, GFP was shown to significantly attenuate this process in all respects, except for ALT levels (Figure 1). These results indicate the ability of GFP to improve the obesity, liver function, and lipid levels of NAFLD rats.

### 2.2. Effect of GFP on the Histopathology of NAFLD Rats

**Figure 2:**
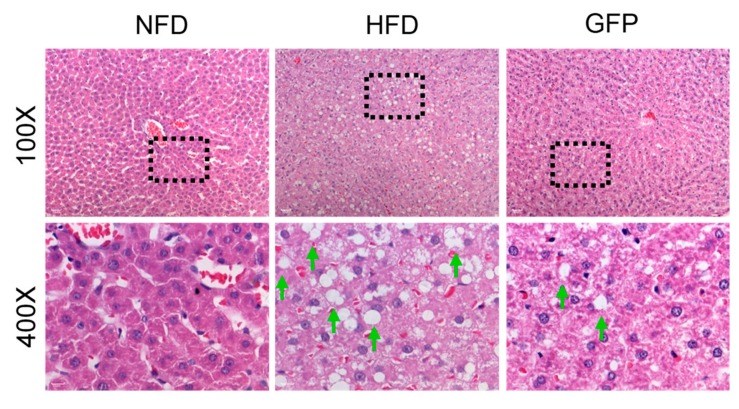
Photomicrographs of rat liver tissues (hematoxylin-eosin staining, 100× and 400×) and histological scores of liver sections. Representative pictures of different groups and higher magnifications of the black dotted boxes in the lower row (green arrows: macrovesicular steatosis).

The histopathological changes in hepatic tissue were examined by light microscopy. As shown in Figure 2, liver sections from normal rats showed distinct hepatic cells with abundant cytoplasm, prominent nuclei, and distinct nucleoli. Neither macrovesicular steatosis nor hepatocellular injury was observed. The hepatic cords radiated in regular rays from the central vein in all directions. In contrast, liver tissues from rats in the HFD group exhibited massive fat droplets in the cytoplasm of hepatocytes, and severe steatosis (>60% of hepatocytes involved) was found in the HFD group, with a high macrovesicular component. However, in the GFP group, steatosis reversal was detected with less than 5% macrovesicular steatosis retained [21,22]. The histological scores of liver sections are presented in Table 2 [23]. The GFP group had significantly reduced hepatocyte steatosis and liver cell injury compared with the HFD group (*p* < 0.01 and *p* < 0.05). However, there was no significant difference in lobular inflammation in the GFP group compared with the HFD group.

### 2.3. Effect of GFP on the mRNA Expression of Genes Involved in NAFLD

**Figure 3:**
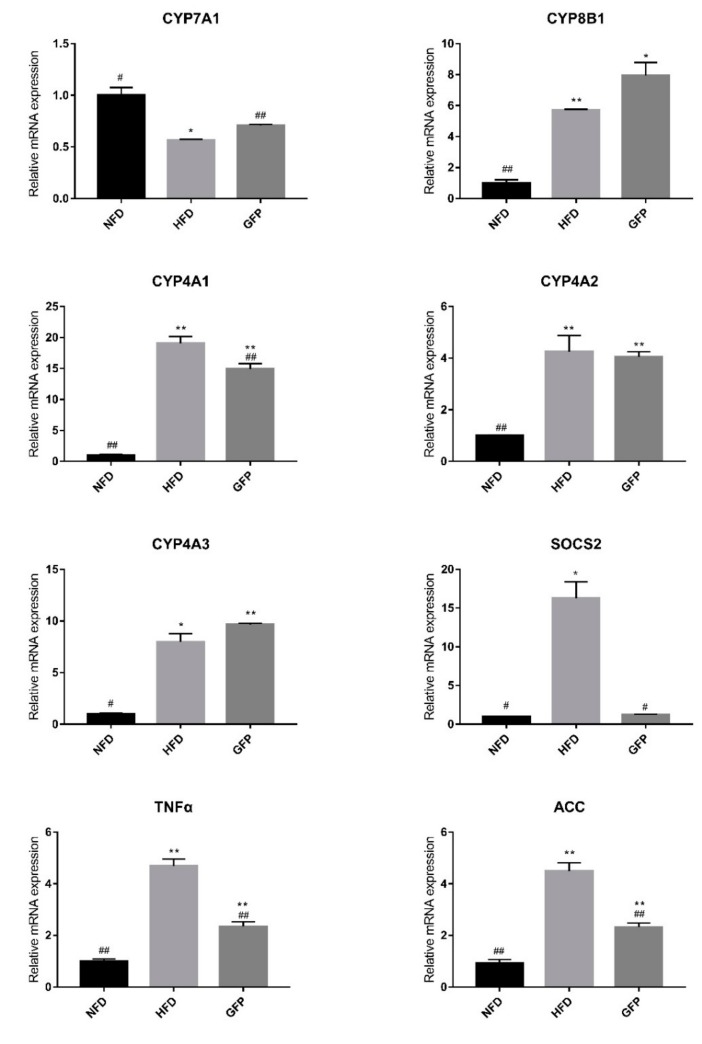
Effect of GFP on the mRNA expression of genes involved in NAFLD as determined by real-time PCR. *β-actin* was used as a reference gene. Data are presented as the mean ± SD, *n* = 3. Differences are denoted on the data labels as follows: * *p* < 0.05 and ** *p* < 0.01 compared with the NFD group; # *p* < 0.05 and ## *p* < 0.01 compared with the HFD group.

To elucidate the molecular mechanism by which GFP ameliorates HFD-induced NAFLD, the hepatic mRNA expression levels of genes involved in lipid metabolism, oxidative stress, and inflammatory cascades were quantified by RT-qPCR. The administration of HFD resulted in elevations in the mRNA levels of *CYP8B1*, *SOCS2*, *CYP4A1*, *CYP4A2*, *CYP4A3*, *TNF-α*, and *ACC* and a reduction in *CYP7A1* gene expression, as shown in Figure 3 (*p* < 0.05). However, GFP treatment significantly reduced the expression of *CYP4A1*, *ACC*, *TNF-α*, and *SOCS2* genes (*p* < 0.05 or *p* < 0.01), and upregulated the expression of *CYP7A1* compared with the HFD group (*p* < 0.01) (Figure 3). Taken together, the above results demonstrate that GFP ameliorates NAFLD by regulating *CYP4A1*, *ACC*, *TNF-α*, *SOCS2*, and *CYP7A1*. These genes play roles in fatty acid oxidation, lipid synthesis, bile acid anabolism, and inflammatory cytokine activity.

### 2.4. Effects of GFP on the Community Structure of the Gut Microbiota in NAFLD Rats

**Figure 4:**
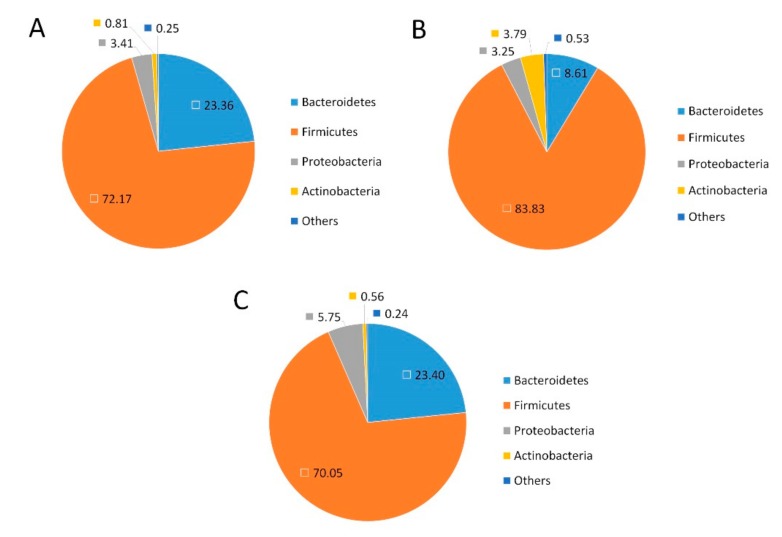
Fecal bacteria at the phylum level based on relative abundance. The color and number correspond to the normalized relative abundance (percent) of the taxa at the phylum level: (**A**) the NFD group; (**B**) the HFD group; and (**C**) the GFP group.

To investigate the effects of the treatments on the community structure of the gut microbiota, the bacterial communities were analyzed at the phylum level (Figure 4). The abundance of Firmicutes was increased, while Bacteroidetes were significantly decreased in the HFD group compared with the NFD group. Compared with the HFD group, the GFP groups exhibited a significant increase in Bacteroidetes and a significant decrease in Firmicutes.

**Figure 5:**
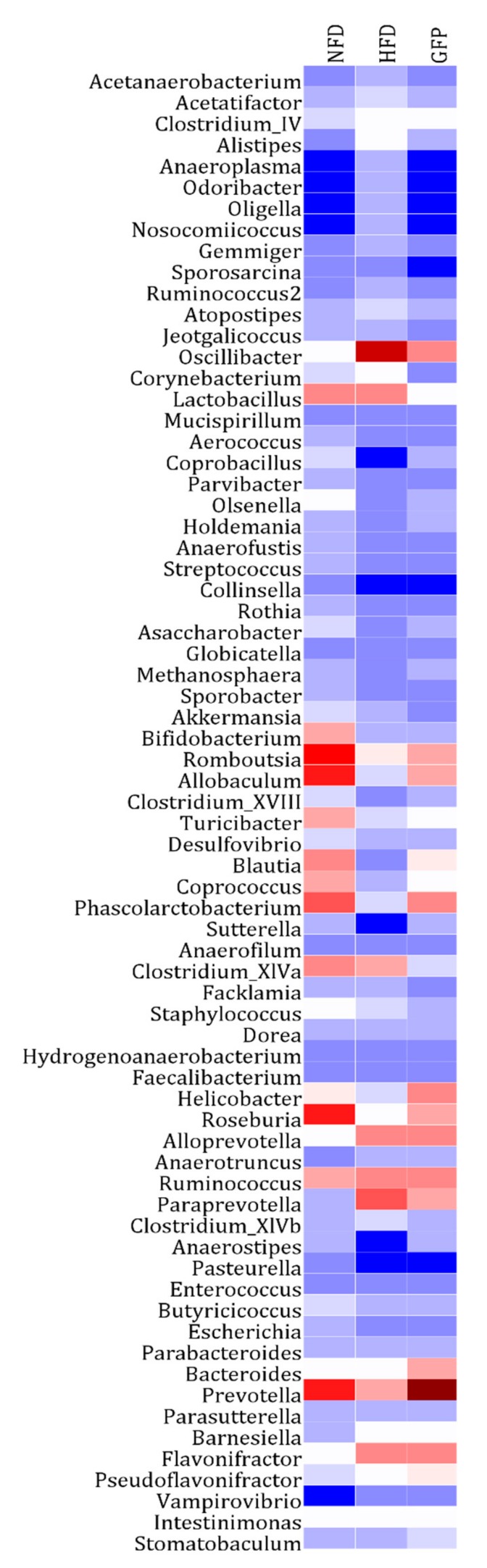
Heatmap of fecal bacterial taxa at the genus level based on relative abundance. The color of each spot corresponds to the normalized relative abundance (percent) of the taxa at the genus level. The genus names of the taxa are shown on the left. The fecal samples from the NFD, HFD, and GFP groups are shown on the top.

The gut microbiota analysis at the genus level revealed HFD-induced changes in the composition of the gut microbiota. Our results show that the relative abundances of *Allobaculum*, *Bacteroides*, *Bifidobacterium*, *Blautia*, *Coprococcus*, *Phascolarctobacterium*, *Prevotella*, and *Roseburia* decreased in the HFD group compared with the NFD group. Conversely, the relative abundances of *Acetatifactor*, *Alistipes*, *Flavonifractor*, *Paraprevotella*, and *Oscillibacter* significantly increased in the HFD group compared with the NFD group. The HFD-induced changes in the cecal microbiota structure were partly reversed by GFP (Figure 5).

**Figure 6:**
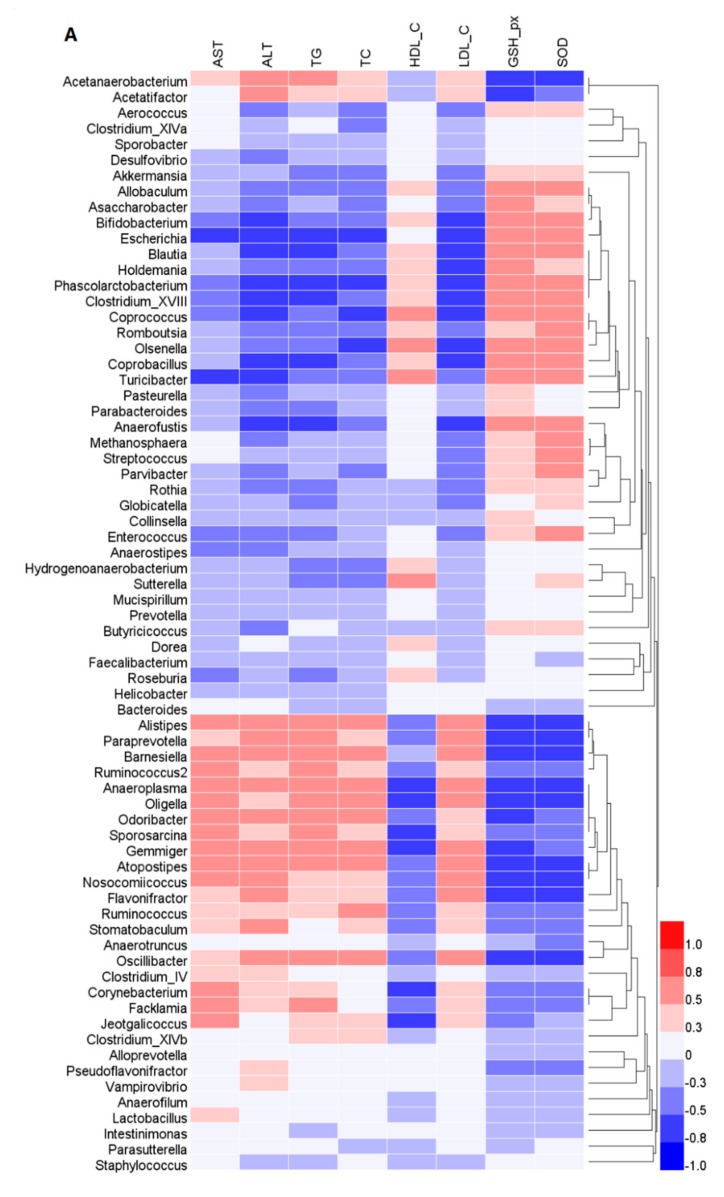

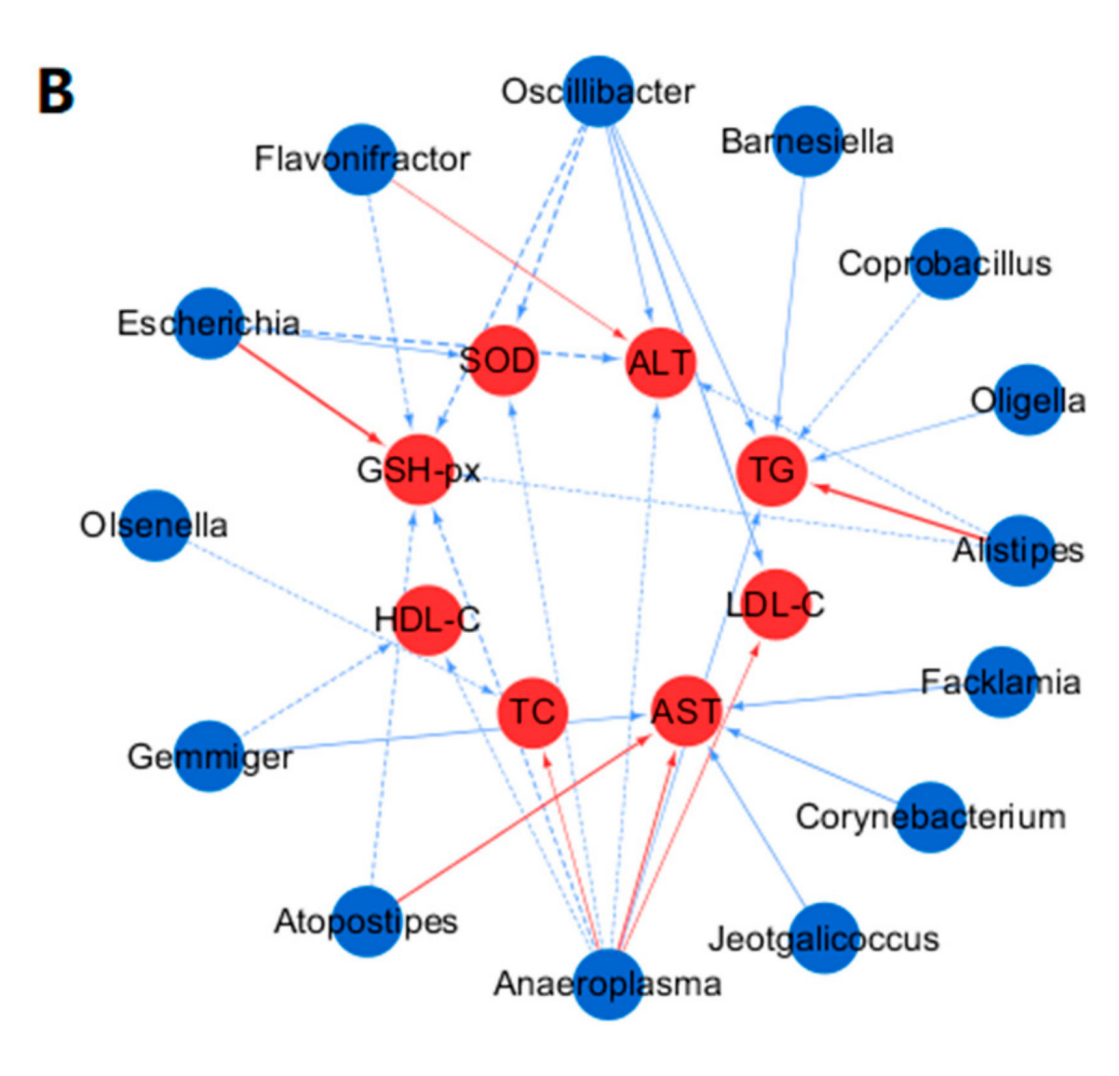
Spearman’s correlations between the cecal microbiota and biochemical parameters measured in this study. (**A**) Heatmap of Spearman’s correlation values showing the significant relationships between cecal microbiota and biochemical parameters. The color intensity represents the degree of association between cecal microbiota and biochemical parameters. (**B**) Visualization of the correlation network according to the significant correlations among the cecal microbiota and biochemical parameters. The significant edges were drawn in the network using the Spearman’s correlation test (|r| > 0.7). Each node represents a gut microbiota genus (blue nodes) and parameters (red nodes). The red and blue lines represent positive and negative correlations, respectively. In addition, the width of the lines indicates the strength of the correlation.

To assess the potential relationship between GFP-induced changes in gut microbiota composition and biochemical indicator levels, Spearman’s correlation analysis was carried out (Figure 6A). The results of Spearman’s correlation analysis showed that the abundance of bacteria belonging to *Acetatifactor*, *Alistipes*, *Flavonifractor*, *Paraprevotella*, and *Oscillibacter*, which were decreased in the GFP compared with the HFD group, were positively correlated with AST, ALT, TC, TG, and LDL-C concentrations, but negatively correlated with GSH-Px, SOD, and HDL-C levels. A visualization of the correlation network based on the significant correlations between the cecal microbiota and the biochemical parameters was constructed (Figure 6B). According to Spearman’s correlation test (|r| > 0.7), significant cecal microbiota, such as *Flavonifractor, Oscillibacter,* and *Alistipes*, are included in the network.

## 3. Discussion

Some studies have suggested that long-term HFD intake leads to TG, TC, and LDL-C synthesis rates exceeding the transport speed and metabolism in hepatocytes, inducing the occurrence of NAFLD [24]. HFD intake has been shown to be important in the development of NAFLD and is associated with increased oxidative stress and hepatic ectopic fat storage [25]. Notably, lipid accumulation in non-adipose tissues can lead to cellular dysfunction and the occurrence of NAFLD [26]. The abnormal oxidation of fatty acids results in reactive oxygen species formation, cell membrane fatty acid and phospholipid disruption, and cholesterol level changes, among other effects [25]. The most striking result of liver injury is the release of intracellular ALT and AST into the bloodstream, resulting in increased serum levels of these enzymes which reflect the extent of liver damage [27]. SOD and GSH-Px play vital roles in scavenging free radicals and maintaining the equilibrium of the redox state by synergistically acting on different parts of the free radical metabolic pathway to prevent oxidative damage [28,29,30].

Considering the side effects associated with many drugs, it is necessary to identify natural product-based drugs to combat NAFLD. Edible fungi, such as *Grifola frondosa*, have traditionally been used to treat human diseases. GFP is a natural heteropolysaccharide extracted from *Grifola frondosa.* In this study, animals administrated with GFP showed an obviously lower body weight when they were fed with HFD after both four and eight weeks (*p* < 0.01) (Table 1). In addition, GFP reduced the concentrations of AST, TC, TG, and LDL-C and increased HDL-C, SOD, and GSH-Px concentrations compared with those of rats in the HFD group in serum or liver homogenates (Figure 1). The histological and pathological observations of the liver showed that GFP reduced hepatocyte steatosis compared with levels present in the HFD group (*p* < 0.01) (Figure 2). The above results indicate that the superior ability of GFP to attenuate NAFLD and obesity may be due to its profound and effective inhibition of fat accumulation induced by HFD.

We also investigated the expression levels of several genes related to oxidative stress, cytochrome P450 activation, bile acid anabolism, and inflammatory cytokine activity to explore the possible mechanism by which GFP may alleviate NAFLD [31,32]. A previous study showed that cytochrome P450 enzymes (CYPs) are associated with lipid peroxidation and damage to the integrity of cells. The transcriptional activation of *CYP4A1* and *CYP4A3* genes in AOX-/- mouse livers could lead to the metabolism of long-chain fatty acids into toxic dicarboxylic acids [33]. Accordingly, *CYP4A2* was shown to be highly expressed in the liver tissues of rats with carbon tetrachloride-induced liver injury [34]. In our study, *CYP4A1*, *CYP4A2*, and *CYP4A3* were found to be highly expressed in the livers of HFD rats, which may have caused liver cell and mitochondrial damage. The GFP group showed downregulation of the expression level of *CYP4A1*, but there was no difference regarding *CYP4A2* and *CYP4A3* expression compared with HFD group. CYP7A1 is the rate-limiting enzyme in the bile acid anabolism pathway, and the other bile acid anabolism pathway is an alternative pathway that is regulated by *CYP8B1* [35]. Previously, the *CYP7A1* mRNA levels of db/db mice or animals fed HFD were shown to be downregulated [35,36]. In our experiment, *CYP7A1* was downregulated in the HFD group compared with the NFD group. In contrast, *CYP7A1* was significantly upregulated in the GFP group compared with the HFD group (*p* < 0.01). The increased *CYP7A1* induced by GFP supplementation may regulate the conversion of cholesterol to bile acids [22]. Moreover, GFP administration effectively reduced the expression of *ACC*, *TNF-α,* and *SOCS2*. The decrease in *ACC* gene expression could promote fat metabolism, thereby reducing TG levels in the liver [37]. TNF-α is considered to be one of the major inflammatory mediators found in NAFLD [38,39]. Compared with healthy subjects, patients with NASH showed overexpression of TNF-α, but inhibition of TNF-α could significantly reduce aminotransferases levels [40]. Therefore, the suppression of TNF-α activation may be one of the mechanisms by which GFP protects hepatocyte membrane structure and function [21,41]. *SOCS2* is an important regulator of hepatic lipid metabolism under HFD conditions. The concentration of TG is much lower in *SOCS2* knockout mice than in wild-type mice that were fed HFD [42]. The reduction in *SOCS2* gene expression following GFP administration shown in our study may be one of the mechanisms which promotes fat metabolism, thereby reducing liver lipid levels.

This study also aimed to determine the effect of GFP on modulating gut microbiota in HFD-fed rats by high-throughput next-generation 16S rRNA gene sequencing. Gut microbes are vital to a host’s health in terms of digesting complex carbohydrates, maintaining energy homeostasis, and regulating immune function, and they are correlated with the development of metabolic disorders, such as NAFLD and diabetes. Notably, the composition of gut microbiota varied after treatment with GFP. Compared with the HFD group, the GFP group showed a decrease in the Firmicutes-to-Bacteroidetes ratio. As reported in a previous investigation, Bacteroidetes, Firmicutes, and Actinobacteria are responsible for the degradation of complex, non-digestible polysaccharides [43]. An increased Firmicutes-to-Bacteroidetes ratio leads to a greater energy-harvesting capacity from undigested carbohydrates, producing more lipids [44]. Therefore, the decrease in liver steatosis observed in the GFP group may be related to the decrease in the Firmicutes-to-Bacteroidetes ratio. Moreover, the relative abandance of *Allobaculum*, *Bacteroides*, *Bifidobacterium*, *Blautia*, *Coprococcus*, *Phascolarctobacterium*, *Prevotella*, and *Roseburia* were significantly increased in the GFP group and the relative abandance of *Acetatifactor*, *Alistipes*, *Flavonifractor*, *Paraprevotella*, and *Oscillibacter* were significantly decreased in the GFP group compared with the HFD group. In addition, *Acetatifactor, Flavonifractor,* and *Oscillibacter* were shown to be significant components of the cecal microbiota according to the Spearman’s correlation test between the gut microbiota and biomedical assays (|r| > 0.7). Therefore, *Acetatifactor*, *Flavonifractor*, and *Oscillibacter* may be very important in the improvement of NAFLD. Some studies have revealed that relative abundance of *Allobaculum*, *Bacteroides*, *Bifidobacterium*, *Prevotella*, and *Roseburia* is associated with a lean phenotype and that these genera are decreased in animals fed HFD [43,44,45,46,47]. A decrease in *Phascolarctobacterium* was correlated with NAFLD and T2D [48]. Moreover, a lower abundance of *Bifidobacterium* was correlated with liver injury and inflammation [46,49]. Some studies have reported that *Roseburia*, *Coprococcus*, and *Blautia* are also beneficial for host health [50,51]. In our study, supplementation with GFP significantly increased the proportions of *Allobaculum*, *Bacteroides*, *Bifidobacterium*, and other cecal microbiota, which may contribute to the beneficial effects of these treatments on the host by boosting the immune system and allowing it to fight against NAFLD. Recent studies have shown that *Acetatifactor*, *Paraprevotella,* and *Oscillibacter* are often increased in response to high-fat or high-sucrose diets [52,53,54]. An increase in *Flavonifractor* is associated with colorectal cancers [55] and displays invasive potential for background inflammatory bowel diseases. *Alistipes* was found to produce an unusual class of bacterial sulfonolipids, which increases in the mouse cecum in response to HFD. These results demonstrate that GFP, as a heteropolysaccharide, is a potential prebiotic that may modulate and maintain the microbial community, thus playing a vital role in relieving NAFLD. The results of this study may also serve as a novel experimental evidence of the ability to prevent and treat NAFLD through the diet. However, the modulation of expression of specific gene members related to lipid synthesis and conversion, cholesterol metabolism, and inflammation pathways were analyzed in this study, and the lack of global gene expression data may be causing some bias. The potential active mechanisms of GFP should be validated in further studies. In addition, the effects of gut microbiota on NAFLD are complex. Thus, the relationship between the gut microbiota and the positive effect of GFP in preventing NAFLD need to be investigated in further studies.

## 4. Materials and Methods

### 4.1. Chemicals and Materials

Fruiting bodies of *Grifola frondosa* were provided by the Qingyuan County of Zhejiang Province (Lishui, China). Chemicals and reagents were purchased from Sinopharm (Shanghai, China). Commercial test kits were obtained from the Jiancheng Bioengineering Company (Nanjing, China).

### 4.2. Preparation of GFP

Dried *Grifola frondosa* was chopped by an electric grinder and sieved through an 80 mesh to prepare dry *Grifola frondosa* powder. *Grifola frondosa* powder (100 g) was pretreated 5 times with (*v*/*w*) 95% ethanol using ultrasonic extraction (300 W, 50 °C) for 60 min. The precipitate was extracted 5 times with (*v*/*w*) 55% ethanol using ultrasonic extraction (300 W, 50 °C) for 60 min. Subsequently, the precipitate was extracted 10 times with hot water (*v*/*w*) using ultrasonic extraction (300 W, 80 °C) for 60 min. The supernatant was again precipitated with the addition of cold absolute ethanol to a final concentration of 80% (*v*/*v*) and kept at 4 °C overnight. The resulting precipitation was vacuum concentrated and freeze dried, yielding *Grifola frondosa* heteropolysaccharide (GFP).

### 4.3. Experimental Animals and Study Design

Male Wistar rats weighing 170 ± 20 g were purchased from Shanghai SLAC Laboratory Animal Co., Ltd (Shanghai, China). The rats were kept under controlled conditions in a temperature range of 25 ± 2 °C and a humidity level of 50 ± 10% with a 12 h light/dark cycle. Rats were provided ad libitum access to commercial rodent chow and water.

After one week of adaptation to the environment, the rats were randomly divided into five groups of eight. Based on our previous preliminary experiments, in addition to water, the animals were given free access to a control diet (NFD, 13.5% energy from fat; Lab Diet 1022; Lab Diet, Beijing, China), a high-fat diet (HFD, 67% control diet, 20% sucrose, 10% lard, and 3% cholesterol), or a high-fat diet with a daily gavage of GFP (150 mg/kg·bw [9]. Body weight was measured at 0, 4, and 8 weeks during treatment. This article does not contain any studies with human participants performed by any of the authors. All procedures involving animals were conducted in strict accordance with the Chinese legislation on the use and care of laboratory animals and were approved by the Committee for Animal Experiments. The guidelines were established by the Institutional Animal Care and Use Committee of 900 Hospital of the Joint Logistics Team (Fuzhou General Hospital of Nanjing Military Command) (IACUC approval No. IACUC-2016-30, approved date: 5 July 2016).

### 4.4. Sample Collection

The rats were anesthetized by an intraperitoneal injection of sodium pentobarbital (50 mg kg^−1^ body weight) at the end of the 8-week experimental period. Then, their abdominal cavity was opened and blood samples were collected by performing cardiac puncture. After 1 h, the samples were centrifuged at 800 *g* for 10 min at 4 °C, and the serum samples were subsequently stored. The livers were removed and rinsed with phosphate buffer saline. Partial tissues from the right lateral lobes of the livers were collected and fixed in 4% formaldehyde for histology analysis. Tissues from other partial livers were dissected, snap frozen in liquid nitrogen, and stored at −80 °C with the serum samples until use for further analysis.

### 4.5. Biochemical Determination

Serum was obtained by centrifugation of the blood samples at 3000 rpm for 15 min. Liver tissues were homogenized in a cold solution of 20 mM Tris–HCl (pH 7.4) (1:10, *w*/*v*). The homogenate was centrifuged for 30 min at 2500 *g* [56]. The activities of serum ALT, serum AST, liver GSH-Px, and liverSOD and the levels of HDL-C, LDL-C, TG, and TC were detected using commercial test kits and instruments following the manufacturer’s instructions.

### 4.6. Histology Analysis

The formalin-fixed livers from the rats were processed according to routine techniques, and 5 μm thick paraffin sections were stained with hematoxylin and eosin for histological analysis [5].

### 4.7. RNA Extraction and Real-Time Polymerase Chain Reaction

Frozen liver tissues were homogenized and total RNA was extracted using TRIzol reagent (Invitrogen) in accordance with the manufacturer’s instructions. Total RNA was extracted from the liver tissues using an RNA extraction kit (Code No. 9108/9109; Takara, Beijing, China). cDNA was synthesized using a PrimeScript™ RT Reagent Kit with gDNA Eraser (Code No. RR047A; Takara). RT-PCR was performed using SYBR Premix Ex Taq II (Code No. RR820A; Takara) in a 25 μL reaction volume. Six selected candidate genes, *CYP7A1*, *CYP8B1*, *CYP4A1*, *CYP4A2*, *CYP4A3*, and suppressor of cytokine signaling 2 (*SOCS2*), tumor necrosis factor alpha (TNF-α), and acetyl-CoA carboxylase (ACC) were assayed by qPCR [57]. The oligonucleotide primers used are shown in Table 3. All reactions were run in triplicate. The conditions for the PCR were 95 °C for 30 s followed by 45 cycles of 95 °C for 5 s, 57 °C for 10 s, and 72 °C for 15 s using an ABI PRISM 7300 thermal cycler.

### 4.8. Cecal DNA Extraction and High-Throughput Sequencing

Cecal DNA was prepared using a QIAamp-DNA Stool Mini Kit (Qiagen, Hilden, Germany) in accordance with the manufacturer’s protocols. The DNA sequences of cecal contents were individually amplified with primer pairs targeting the 16S rRNA gene. The V3–V4 region of the 16S rRNA gene (V3–V4 hypervariable regions) was amplified by PCR using specific primers (forward primer: 5′-CCTACGGRRBGCASCAGKVRVGAAT-3′; reverse primer: 5′-GGACTACNVGGGTWTCTAATCC-3′). High-throughput sequencing was completed by Itechgene Technology Co., Ltd. (Shanghai, China).

### 4.9. Statistical Analysis

Statistical analysis was performed using one-way analysis of variance (ANOVA) and multiple comparisons were carried out to test for any significant differences among three groups with the SPSS software package, version 13.0. Values from each group were expressed as the mean ± SD. *p* < 0.05 and *p* < 0.01 were considered to indicate statistical significance. The relative abundance of gut flora in different groups of rats was compared using R software (http://www.r-project.org/).

## Figures and Tables

**Table 1:** Effects of GFP on body weight and body weight gain as compared withNFD and HFD.

Time	Weight (g)		
	NFD	HFD	GFP
0 weeks	222.86 ± 11.1	230.79 ± 5.93	229.98 ± 8.03
4 weeks	376.75 ± 31.65	409.78 ± 27.53 *	362.1 ± 27.67 ^##^
8 weeks	388.74 ± 30	442.73 ± 33.22 **	384.31 ± 30.97 ^##^
weight gain	165.88 ± 22.05	211.95 ± 32.88 **	154.33 ± 32.75 ^##^

Values are presented as the mean ± SD (*n* = 8). Differences were assessed by ANOVA and are denoted as follows: * *p* < 0.05 versus the NFD group; ** *p* < 0.01 versus the NFD group; and ## *p* < 0.01 versus the HFD group.

**Table 2:** The histological scores of liver sections of GFP group as compared with NFD and HFD groups.

Histological Scores	Steatosis Grade	Lobular Inflammation	Liver Cell Injury Ballooning
NFD	0 ± 0	0 ± 0	0.4 ± 0.55
HFD	2.4 ± 0.55 **	0.4 ± 0.55	2 ± 0 *
GFP	0.8 ± 0.45 ^##^	0.2 ± 0.45	1.6 ± 0.55 *

Values are presented as the mean ± SD (*n* = 5). Differences were assessed by ANOVA and are denoted as follows: * *p* < 0.05 versus the NFD group; ** *p* < 0.01 versus the NFD group; and ## *p* < 0.01 versus the HFD group.

**Table 3:** Sequences of primers used for RT-PCR in this study. *ACC*: acetyl-carboxylase; *TNF-α*: tumor necrosis factor alpha.

Gene	Forward Primer	Reverse Primer
*β-actin*	5′-GGCACCACACTTTCTACAAT-3′	5′-AGGTCTCAAACATGATCTGG-3′
*CYP7A1*	5′-GAGGGATTGAAGCACAAGAACC-3′	5′-ATGCCCAGAGAATAGCGAGGT-3′
*CYP8B1*	5′-CCCCTATCTCTCAGTACACATGG-3′	5′-GACCATAAGGAGGACAAAGGTCT-3′
*CYP4A1*	5′-TTGAGCTACTGCCAGATCCCAC-3′	5′-CCCATTTTTGGACTTCAGCACA-3′
*CYP4A2*	5′-CTCGCCATAGCCATGCTTATC-3′	5′-CCTTCAGCTCATTCATGGCAATT-3′
*CYP4A3*	5′-CTCGCCATAGCCATGCTTATC-3′	5′-CCTTCAGCTCATTCATGGCAATC-3′
*SOCS2*	5′-GGAACGGCACTGTTCACCTTTA-3′	5′-AGCCTACAGAGATGCTGCAGAGA-3′
*TNF-α*	5′-TGAACTTCGGGGTGATCGGT-3′	5′-CTCCTCCGCTTGGTGGTTTG-3′
*ACC*	5′-ACACTGGCTGGCTGGACAG-3′	5′-CACACAACTCCCAACATGGTG-3′
